# Disruption of the Glutamate–Glutamine Cycle Involving Astrocytes in an Animal Model of Depression for Males and Females

**DOI:** 10.3389/fnbeh.2016.00231

**Published:** 2016-12-06

**Authors:** Virginie Rappeneau, Amanda Blaker, Jeff R. Petro, Bryan K. Yamamoto, Akiko Shimamoto

**Affiliations:** ^1^Department of Neuroscience and Pharmacology, Meharry Medical College School of MedicineNashville, TN, USA; ^2^Department of Pharmacology and Toxicology, Indiana University School of MedicineIndianapolis, IN, USA

**Keywords:** anhedonia, astrocytes, chronic social defeat stress, glial fibrillary acidic protein, glutamate–glutamine cycle, glutamate transporter-1, sex

## Abstract

**Background:** Women are twice as likely as men to develop major depression. The brain mechanisms underlying this sex disparity are not clear. Disruption of the glutamate–glutamine cycle has been implicated in psychiatric disturbances. This study identifies sex-based impairments in the glutamate–glutamine cycle involving astrocytes using an animal model of depression.

**Methods:** Male and female adult Long-Evans rats were exposed to chronic social defeat stress (CSDS) for 21 days, using a modified resident-intruder paradigm. Territorial aggression was used for males and maternal aggression was used for females to induce depressive-like deficits for intruders. The depressive-like phenotype was assessed with intake for saccharin solution, weight gain, estrous cycle, and corticosterone (CORT). Behaviors displayed by the intruders during daily encounters with residents were characterized. Rats with daily handling were used as controls for each sex. Ten days after the last encounter, both the intruders and controls were subjected to a no-net-flux *in vivo* microdialysis to assess glutamate accumulation and extracellular glutamine in the nucleus accumbens (NAc). The contralateral hemispheres were used for determining changes in astrocytic markers, including glial fibrillary acidic protein (GFAP) and glutamate transporter-1 (GLT-1).

**Results:** Both male and female intruders reduced saccharin intake over the course of CSDS, compared to their pre-stress period and to their respective controls. Male intruders exhibited submissive/defensive behaviors to territorial aggression by receiving sideways threats and bites. These males showed reductions in striatal GLT-1 and spontaneous glutamine in the NAc, compared to controls. Female intruders exhibited isolated behaviors to maternal aggression, including immobility, rearing, and selfgrooming. Their non-reproductive days were extended. Also, they showed reductions in prefrontal and accumbal GFAP^+^ cells and prefrontal GLT-1, compared to controls. When 10 μM of glutamate was infused, these females showed a significant accumulation of glutamate compared to controls. Infusions of glutamate reduced extracellular glutamine for both male and female intruders compared to their respective controls.

**Conclusion:** Twenty-one days of territorial or maternal aggression produced a depressive-like phenotype and impaired astrocytes in both male and female intruders. Disruption of the glutamate–glutamine cycle in the PFC-striatal network may be linked to depressive-like deficits more in females than in males.

## Introduction

Major depression (MD) is a female-predominant psychiatric illness (Piccinelli and Wilkinson, 2000). One cause of this sex difference is the dysfunction of female gonadal hormones, including estrogens (Bloch et al., 2000; Cohen et al., 2006; Freeman et al., 2006). In fact, supplement of estrogens can improve some symptoms of MD and can also enhance the actions of antidepressants (Martenyi et al., 2001; Thase et al., 2005; Berlanga and Flores-Ramos, 2006). However, the efficacy of estrogen administration or antidepressants seems to vary across female MD patients, regardless of their reproductive stages (Martel et al., 2009). Therefore, it is warranted to investigate factors other than the dysfunction of the hypothalamic-pituitary-gonadal (HPG) axis in the sex disparity of MD.

Stressful life events are the most common factor for the development and recurring symptoms of MD in both men and women (Kendler et al., 1999; Slavich and Irwin, 2014). Some cardinal features of human depression, such as anhedonia and loss of appetite, can be modeled in laboratory animals by repeatedly exposing them to stress. Social defeat stress refers to an animal receiving constant attacks by means of territorial, offensive, or defensive aggression from a conspecific same sex animal; namely a resident-intruder paradigm (Koolhaas et al., 2011; Miczek et al., 2011b; Takahashi and Miczek, 2014). A daily social defeat ranging from 10 days to 5 weeks produces anhedonia-like responses, reduces locomotor activity, and induces social avoidance that can be reversed by chronic pharmacological antidepressant treatments (Berton and Nestler, 2006; Tsankova et al., 2006; Rygula et al., 2008). Hence, the chronic social defeat stress (CSDS) justifies face, construct, and predictive validity of this model of depression, particularly when tested in males (Anisman and Matheson, 2005; Nestler and Hyman, 2010; Golden et al., 2011; Dzirasa and Covington, 2012). By contrast, few studies have developed a female model of depression (Chaouloff, 2013; Hammels et al., 2015). Previously, we have shown that female social defeat stress by use of maternal aggression, in which a lactating dam shows defensive aggression toward unfamiliar intruders (de Almeida et al., 2014), induces anhedonia-like responses, disrupts estrous cycle, and alters dopaminergic and behavioral responses to psychostimulants, including cocaine (Miczek et al., 2011a; Shimamoto et al., 2011, 2014).

The glutamate–glutamine (Glu–Gln) cycle refers to the transport of glutamate and its amidated molecule glutamine in the tripartite synapse in the brain (Broer and Brookes, 2001; Bak et al., 2006; Albrecht et al., 2007). Once released from presynapses, extracellular Glu is transported to surrounding glial cells, including astrocytes, via glutamate transporter-1 (GLT-1). Glu is then amidated to Gln, a non-toxic neutral amino acid. A removal of Glu from extracellular space (ECS) by astrocytes maintains the homeostasis of glutamate in the tripartite synapse. If astrocytes do not function properly, the glutamate molecules accumulate in the ECS, leading to glutamate excitotoxicity (Rothstein et al., 1996). Animal studies show that dysfunction of GLT-1 can induce some depressive-like behaviors, and drugs that upregulate GLT-1 ameliorate such deficits (Banasr and Duman, 2008; Banasr et al., 2010). In addition, cell culture studies show that estrogens enhance GLT-1 activity and accelerate glutamate uptake by facilitating GLT-1 promotor activity (Pawlak et al., 2005b; Lee et al., 2013; Karki et al., 2014). Taken together, the Glu–Gln cycle involving astrocytes may play a role in mediating depressive-like deficits when exposed to stress repeatedly.

In this study, we identified sex-based disruption in the Glu–Gln cycle involving astrocytes targeting the PFC-NAc pathway (Nestler and Carlezon, 2006; Nestler, 2015), using an animal model of depression for males and females.

## Materials and Methods

### Animals

Male (225–250 g, *n* = 51) and female (200–225 g, *n* = 72) Long-Evans rats (Charles River Laboratories, Raleigh, NC, USA) were individually housed in rat standard cages in an environmentally controlled vivarium (21 ± 1°C; 30–70% humidity; inverted 12-h light–dark cycle) and with *ad libitum* access to food and water. Separate “resident” male and female rats (>300 g) were paired in guinea pig cages (27 cm × 51 cm × 22 cm) located in a separate vivarium. All animal protocols were carried out in accordance with the National Institutes of Health Guide for Care and Use of Laboratory Animals and were approved by the Institutional Animal Care and Use Committee of Meharry Medical College (National Research Council, 2011).

### Drugs

All drugs and chemicals were purchased from Sigma-Aldrich (St. Louis, MO, USA), except for artificial cerebrospinal fluid (aCSF) (Harvard Apparatus, Holliston, MA, USA), methanol (EMD Millipore, Billerica, MA, USA), ethylenediamine-*N,N,N*′*,N*′-tetraacetic acid, disodium salt, dehydrate (EDTA ∙ 2Na) (Dojindo Molecular Technologies, Inc., Kumamoto, Japan), 1X Halt protease inhibitor cocktail (Cat. 78430, Thermo Scientific, Rockford, IL, USA), Novex 4X LDS sample buffer (Invitrogen, Carlsbad, CA, USA), and HyGlo enhanced chemiluminescence (ECL) (Denville Scientific, Inc., Metuchen, NJ, USA).

### Saccharin Intake

Intake for saccharin was measured at least three times a week during the pre-stress period (1 week) and during 3 weeks of CSDS to assess the rats’ anhedonia-like responses (Shimamoto et al., 2011, 2014). Rats were presented with two bottles containing either a 0.02% saccharin solution or tap water for 60 min from 10 to 11 AM. The position of bottles were counterbalanced to avoid side preference. Rats that had saccharin intake outside of mean ± 1 SD within a cohort during the pre-stress period were excluded from further experiments (4 males and 13 females).

### Estrous Cycle

Estrous cycle was determined by cytological examination of vaginal smears, using the Giemsa staining method (Staples and Geils, 1965; Shimamoto et al., 2011, 2014). Estrous phase was assigned to reproductive (proestrus and estrus, and a transition phase from proestrus to estrus) or non-reproductive (late estrus, metestrus, diestrus, and any transition phases between these phases) days. Any rats that had more than three or no reproductive days during the pre-stress period were excluded from further experiments (two females).

### Screening for Candidate “Residents”

#### Males

Candidate male “resident” rats were paired with females for at least 3 weeks before being screened to establish territorial aggression toward unfamiliar young male intruders (Tornatzky and Miczek, 1993; Miczek et al., 2011a). The candidates that showed stable territorial and offensive behaviors, based on criteria including latency to first attack bite (<5 min) and number of attack bites during a screening session for at least three consecutive sessions, were subsequently used as “residents” to induce social defeat stress to male intruders described below.

#### Females

Lactating dams whose pups were 3–12 days old were used as aggressive “residents” (Shimamoto et al., 2011, 2014). Unlike male candidates, lactating dams reliably show defensive aggression toward unfamiliar female intruders without being screened (Erskine et al., 1978). However, a loss of pups reduces aggressiveness toward intruders (Lonstein and Gammie, 2002). Therefore, any dams whose pups were not properly fed or died were discontinued from the CSDS paradigm.

### Experimental Design

**FIGURE 1:**
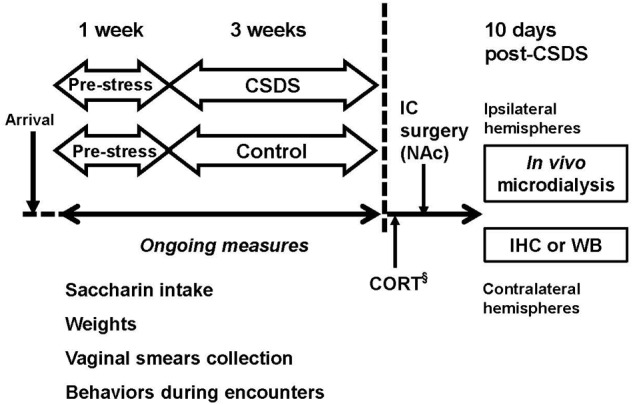
**Experimental design.** Male and female Long-Evans rats underwent 3 weeks of chronic social defeat stress (CSDS), using a modified resident-intruder paradigm. Rats that were used as controls for each sex were handled daily for 21 days. ^§^Separate cohorts of animals used for corticosterone (CORT) measurement. IC, intracranial; IHC, immunohistochemistry; WB, Western Blotting.

**Figure 1** shows the experimental design for the study. Male and female rats underwent 1 week of baseline assessment (pre-stress), followed by 3 weeks of CSDS or daily handling (control) described below. Intake for 0.02% saccharin solution, weight, and estrous cycle were monitored during the entire period. Following the exclusion of rats described above, 35 males and 42 female rats were randomly assigned to CSDS or control groups. Direct encounters were filmed for subsequent behavioral characterization of intruders, described below. After the completion of CSDS, all rats underwent intracranial surgery for the no-net-flux *in vivo* microdialysis. The microdialysis experiment took place approximately 10 days after the final social defeat. Twenty-four hours after the *in vivo* microdialysis experiment, the contralateral hemispheres were collected for analyses of glial fibrillary acidic protein (GFAP) and GLT-1. Separate cohorts of animals (15 males and 12 females) were used for corticosterone (CORT) assays.

### Chronic Social Defeat Stress

#### Males

The CSDS protocol was a cycle of an encounter between a territorial male resident and a male intruder in the resident’s homecage, followed with a prolonged threat to the intruder by the resident through a custom-built meshed divider (20 cm × 30 cm × 20 cm) (Alternative Design Manufacturing, Siloam Springs, AR, USA) (Miczek, 1979; Miczek et al., 2011a). Before each encounter, the paired female was removed from the male resident’s homecage. On CSDS day 1, a male intruder was introduced into the territorial resident’s homecage and the encounter occurred. After the first attack bite initiated by the resident, the encounter period elapsed either for 5 min or until the intruder showed a 6-s full supine posture, whichever came first. After the direct encounter, the meshed divider was inserted into the cage, and the intruder remained in one compartment while the resident remained in the other for the remainder of 24 h. This housing arrangement allowed sensory contact and threat from the resident, while protecting the intruder from any additional injury. On the next day, the intruder was removed from the resident’s homecage and placed in an unfamiliar resident’s homecage for a new encounter. This encounter-threat cycle was repeated for 21 days. One male was removed from the study due to a severe wound caused by a resident.

#### Females

The CSDS for females was a cycle of an encounter between a lactating “resident” and a female intruder in the lactating resident’s homecage, followed with a prolonged threat to the intruder by the resident through the custom-built meshed divider (Shimamoto et al., 2011, 2014). Before each encounter, the paired male was removed from the lactating resident’s homecage. On CSDS day 1, a female intruder was introduced to the lactating resident’s homecage, and the encounter elapsed for 30 min. This session took place approximately at 12 PM. The encounter was terminated by inserting the divider, which separated the female intruder from the lactating resident. The intruder remained in one compartment while the lactating resident in the other until the next session. Approximately 3 h later, the female intruder was removed from the lactating resident’s homecage and placed in an unfamiliar lactating resident’s homecage for a new encounter. The encounter was followed with the insertion of the divider, and both the intruder and the resident remained there for the remainder of 18 h. Hence, the encounter for females took place for two times a day. This daily regimen continued for 21 days. Vaginal smears were collected from female intruders immediately before placing them to the first encounter of a day (11 AM). All encounters took place in the presence of pups to maintain the aggressiveness of lactating dams (Lonstein and Gammie, 2002). Eight female intruders were discontinued from the CSDS regimen due to a shortage of lactating dams who lost their pups during this period.

After the completion of CSDS, both male and female intruders were returned to their homecage for further experiments described below. Male and female rats who were individually housed and handled daily throughout the 21 days of CSDS in a separate vivarium were used as controls.

### No-Net-Flux *In vivo* Microdialysis

Approximately 2–3 days after the last encounter or the daily handling, 34 males and 34 females underwent stereotaxic surgery under ketamine (100 mg/kg) and xylazine (6 mg/kg for males and 3 mg/kg for females) anesthesia for implantation of a unilateral guide cannula (CXG-8, Eicom USA, San Diego, CA, USA) aimed at the NAc (AP, +1.4 mm from bregma; ML, ±0.9 mm from bregma; and DV, -5.8 mm from dura) (Miczek et al., 2011a; Shimamoto et al., 2011, 2014). On the test day, a 2-mm active membrane probe (CX-I-8-02, Eicom USA, San Diego, CA, USA) was inserted into the guide cannula and was infused with aCSF at a rate of 1.5 μL/min. After an hour of equilibration, microdialysates were collected every 10 min using a refrigerated (4°C) fraction collector (CMA/142, CMA Microdialysis AB, Holliston, MA, USA). The aCSF containing 0, 2.5, 5.0, or 10.0 μM of glutamate was infused into the NAc, with at least 30 min of intervals between the different glutamate concentrations (Baker et al., 2003; Shen et al., 2014). At the end of the experiment, vaginal smears were collected for females to determine the estrous phase of the day. Twenty-four hours later, rats were deeply anesthetized with 50 mg/kg pentobarbital for verification of probe placements. A total of 11 males and 5 females were excluded from the dialysis study due to misplacements of the probe. Within these animals excluded from the microdialysis study, two males and one female had their microdialysis probe in the lateral ventricle. This means that these three rats may have had glutamate infused to contralateral hemispheres through the ventricles. Therefore, these animals were not used for GFAP or GLT-1 experiments.

### Glutamate and Glutamine Assay

Glutamate and glutamine were analyzed using the ortho-phthalaldehyde (OPA) method according to manufacturer’s instruction (Eicom USA, San Diego, CA, USA)^1^. Briefly, 4 mM of OPA-2ME was prepared by a dilution of 20 mM OPA using 0.5 M K_2_CO_3_ containing 0.2% of 2-mercaptoethanol (2-ME). Two and a half μL of 4 mM OPA-2ME was added to a 10 μL of microdialysate and left for 2.5 min, then 10 μL of conjugate was injected onto the high-performance liquid chromatograph (HPLC, HTEC-500) equipped with an autosampler system (AS-700) (Eicom USA, San Diego, CA, USA). The derivatized samples were detected using an electrochemical detector. The HPLC conditions were as follows: separation column Eicompak FA-3ODS (3.0 mm, i.d. x 50 mm, Eicom USA, San Diego, CA, USA); pre-column, CA-ODS (3 mm, i.d. x 4 mm, Eicom USA, San Diego, CA, USA); mobile phase: 0.1 M phosphate buffer (PB) (pH 6.0)-methanol-acetonitrile (80:7:13, v/v) containing 5 mg/L EDTA•2Na; flow rate 500 μL/min; column temperature 40°C. Glutamate and glutamine concentrations were determined using standard curves with known amounts of amino acids in the range of 0.625–10 μM. Under these conditions, the limits of detection for glutamate and glutamine were 0.14 and 0.016 μM, respectively. Samples from one male were excluded from the analysis due to a malfunction of the HPLC.

### GFAP Immunoreactivity (GFAP-IR)

Twenty-four hours after the *in vivo* microdialysis experiment, 11 males and 12 females rats were deeply anesthetized with sodium pentobarbital (50 mg/kg, i.p.) and intracardially perfused with 0.9% saline followed by 4% paraformaldehyde (PFA) in 0.1 M PB. Contralateral hemispheres were immersed overnight in 4% PFA in 0.1 M PB followed by 30% sucrose/PB for cryoprotection. The hemispheres were stored in -20°C until the brain sectioning. Thirty μm of brain slices were prepared using a cryostat machine (Ultrapro 5000, Bright Instruments, Ltd, Bedfordshire, UK). The slices were incubated with rabbit polyclonal primary anti-GFAP antibody (1:2000, ZO334, Dako, Denmark) at 4°C for 3 days, followed by a standard avidin-biotin-peroxidase staining procedure (Vector Laboratories, Inc., Burlingame, CA, USA). DAB was used for revelation. The slices were then counterstained with cresyl violet, dehydrated (alcohol and xylene), and coverslipped with Depex. Normal goat serum and the absence of primary antibodies were used as negative controls. The numbers of GFAP^+^ and Nissl^+^ cells in the NAc, prefrontal cortex (PFC), basolateral and central amygdala, BNST, and dorsal and ventral hippocampus were determined using a Nikon TE2000-E wide-field microscope equipped with a CoolSNAP HQ2 camera connected to a computerized image analysis system (Nikon NIS Elements, Melville, NY, USA). A GFAP^+^ cell was defined as an astrocyte when more than two stem branches extend from a cell body. The average densities of GFAP^+^ and Nissl^+^ cells for each region were determined by analyzing six hemi-sections within the region across the rats. Rat brain atlas was used to determine the regions of interest (Paxinos and Watson, 2013).

### Western Blotting

The modified Western blotting protocol was used (Northrop and Yamamoto, 2012). The PFC, dorsal striatum (Str), and the NAc from contralateral hemispheres from 18 males and 15 females were dissected and homogenized in RIPA buffer (0.1 M PBS, 1% Igepal, 0.5% sodium deoxycholate, and 0.1% sodium dodecyl sulfate) with 1X Halt protease inhibitor cocktail. Protein concentration was determined using a Bradford assay (Bio-Rad Laboratories, Inc., Hercules, CA, USA) and samples were diluted 1:4 with Novex 4X LDS sample buffer. Samples were then heated to 80°C for 5 min. Proteins were loaded for GFAP (20 μg) or glutamate transporter 1 (GLT-1) (5 μg) onto NuPAGE Novex 4–12% Bis-Tris gels (Invitrogen, ThermoFisher Scientific, Inc., Waltham, MA, USA) for electrophoresis. Proteins were transferred onto a PVDF membrane and blocked for 1 h at room temperature with Tris-buffered saline (TBS) (10 mM Tris, 150 mM NaCl), containing 0.5% Tween-20 and 5% non-fat powdered milk. Membranes were incubated in either rabbit anti-GFAP (1:2000, AB5804, EMD Millipore, Billerica, MA, USA) or guinea pig anti-glutamate transporter (1:1000, AB1783, EMD Millipore, Billerica, MA, USA) overnight at 4°C in blocking buffer. Membranes were then washed twice for 3 min in TBS containing Tween-20 (TBS-T) and incubated with horseradish peroxidase (HRP)-conjugated secondary antibodies (goat anti-rabbit IgG, 1:3000, AP307P, EMD Millipore, Billerica, MA, USA or mouse anti-guinea pig IgG, 1:3000, sc-2438, Santa Cruz Biotechnology, Inc., Santa Cruz, CA, USA) in blocking buffer for 1 h at room temperature. After two additional 3 min washes, the membranes were incubated in HyGlo ECL for 2 min for antibody detection. Images were captured via a Fuji LAS-4000 mini system (FujiFilm, Corp., Life Science Division, Tokyo, Japan), and optical density was determined via Multi Gauge software (FujiFilm, Corp., Life Science Division, Tokyo, Japan). GFAP and GLT-1 were both normalized to actin as the internal loading control. GFAP membranes were stripped before probing for actin, as the two proteins have similar molecular weights. Briefly, membranes were incubated in diluted ReBlot Plus Mild Antibody Stripping Solution (EMD Millipore, Billerica, MA, USA) for 20 min at room temperature, washed with blocking buffer three times for 3 min each, blocked for 1 h at room temperature, and then incubated in mouse anti-actin (1:3000, MAB1501, EMD Millipore, Billerica, MA, USA) overnight at 4°C. After two 3 min washes, membranes were incubated in goat anti-mouse IgG (1:3000, sc-2005, Santa Cruz Biotechnology, Inc., Santa Cruz, CA, USA) for 1 h at room temperature in blocking buffer, and developed via ECL.

### Corticosterone (CORT) Assay

After 3 weeks of CSDS using the same protocol described above, rats were deeply anesthetized with pentobarbital (50 mg/kg, i.p.) and trunk blood was collected from the inferior vena cava. Plasma CORT levels were determined using a competitive enzyme immunoassay using manufacturer’s instruction (Corticosterone EIA kit, Cayman Chemical, Co., Ann Arbor, MI, USA). The assay range is between 8.2 and 5,000 pg/mL.

### Behavioral Characterization of Intruders during Encounters

Behaviors displayed by male and female intruders during daily encounters were filmed with a digital video camera (Sony Handycam HDR-PJ340, Sony Corporation of America, New York, NY, USA) and were characterized using The Observer XT software (v. 12.5, Noldus Information Technology, Wageningen, The Netherlands). Individual behaviors characterized are listed in **Table 1** (Huhman et al., 2003; Miczek et al., 2011b). Any behaviors other than these were defined as non-specific behaviors. The characterized behaviors were further collapsed to three categories: submissive/defensive, social orienting/investigating, and isolated behaviors. Encounters recorded on the first 3 days for each week (i.e., CSDS days 1–3, 8–10, and 15–17) were used for characterization.

**Table 1:** Behaviors displayed by intruders during daily encounters (duration, Mean ± SEM, 3-week average).

	Male (5 min maximum)	Female (30 min)
	
Behaviors displayed by intruders	Duration (sec)	
**Submissive/defensive**
Twitching	4.08 ± 1.86	0.0 ± 0.00
Attempted supine posture (<6 s)	19.7 ± 6.47	3.1 ± 2.12
Upright posture	10.6 ± 2.90	0.6 ± 0.44
Escape	1.8 ± 0.47	0.3 ± 0.25
**Social orienting/investigating**
Being sniffed (body and genital)	28.7 ± 2.82	30.8 ± 3.68
Approach to nest, litter, or dam	N/A	34.0 ± 11.67
Allo-grooming	0.0 ± 0.00	46.9 ± 10.96
**Isolated**
Immobile	79.9 ± 21.15	258.9 ± 59.37
Rearing	11.0 ± 3.05	107.2 ± 21.56
Self-grooming	0.0 ± 0.00	84.6 ± 14.86
Digging/foraging	0.0 ± 0.00	14.4 ± 3.76

### Statistical Analyses

SPSS for Windows version 23 (IBM, Armonk, NY, USA) was used for all statistical analyses. Two-way repeated measures ANOVA was used to analyze repeated observations of body weight and intake change for saccharin solution within the CSDS and the controls for each sex; when indicated by a significant main effect, *post hoc* comparisons to the control groups or to the pre-stress baseline were performed using the Holm–Sidak multiple comparisons. An independent Student’s *t*-test was used for changes in non-reproductive days between female CSDS and controls, CORT between CSDS and controls for each sex, GFAP-IR and protein levels and GLT-1 protein levels between CSDS and controls for each sex and each brain region. For the no-net-flux *in vivo* microdialysis study, glutamate accumulation in the NAc was calculated by the difference between various concentrations of glutamate infused (Glu _[In]_) and the concentrations collected from the NAc (ΔGlutamate). Two-way repeated measures ANOVA was used for glutamate accumulation and extracellular glutamine in the NAc for each sex; when indicated by a significant main effect, *post hoc* comparisons to the respective controls using Holm–Sidak corrections were performed for multiple comparisons. Data were expressed as mean ± SEM. *p* values less than 0.05 were considered as significant. The number of animals used in each experiment is listed in Supplementary Table S1 and in Figure Legends.

## Results

### Physiological and Endocrine Changes over the Course of CSDS

**FIGURE 2:**
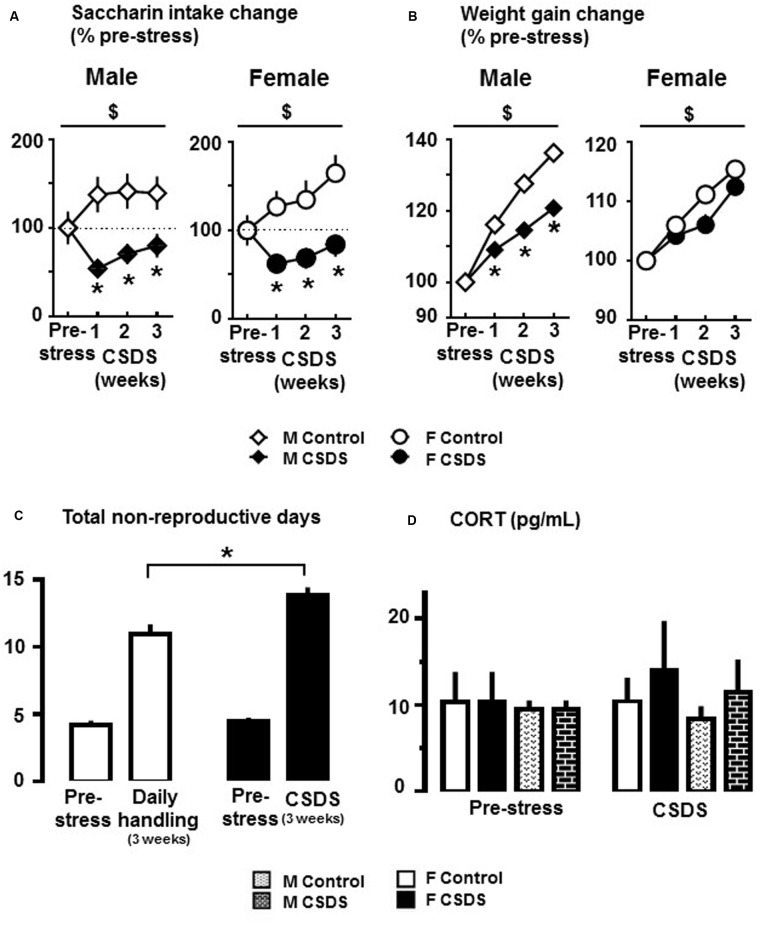
**Anhedonia-like responses and endocrine changes in rats exposed to CSDS. (A)** Changes of 0.02% saccharin solution consumption in CSDS (male, *n* = 19; female, *n* = 16) and control (male, *n* = 15; female, *n* = 18) groups. Data are expressed as % change from saccharin intake during the pre-stress period. **(B)** Average change in body weight gain from pre-stress body weight. Two-way repeated measures of analysis of variance, followed by Holm–Sidak’s *post hoc* test. ^$^Time effect, ^∗^Stress effect. *p* < 0.05. **(C)** Number of non-reproductive days in CSDS (*n* = 16) and control (*n* = 18) females during the pre-stress (sum of 7 days), and CSDS or daily handling (sum of 21 days) periods. Independent *t*-test, one-tailed ^∗^*p* < 0.05 vs. respective control group. **(D)** Corticosterone (CORT) levels (pg/mL) at the end of the pre-stress and CSDS periods in CSDS (male, *n* = 4; female, *n* = 7) and control (male, *n* = 4; female, *n* = 4) groups. Mean ± SEM.

**Figure 2A** shows saccharin intake change in males and females who were exposed to CSDS or daily handling (controls). There were significant main effects of “Time” (*F*_(3,30)_ = 1.744, *p* < 0.05) and “Stress” (*F*_(1,32)_ = 35.929, *p* < 0.05), and a “Time” × “Stress” interaction (*F*_(3,30)_ = 11.519, *p* < 0.05) in males. Also, significant main effects of “Time” (*F*_(3,27)_ = 6.962, *p* < 0.05) and “Stress” (*F*_(1,29)_ = 27.104, *p* < 0.05), and a “Time” × “Stress” interaction (*F*_(3,27)_ = 4.229, *p* < 0.05) were observed in females. All multiple comparisons revealed that saccharin intake was significantly reduced in week 1 of CSDS in both males and females compared to their respective pre-stress period (*p* < 0.05). Also, all pairwise multiple comparisons revealed that saccharin intake was significantly reduced in both CSDS males and females compared to their respective controls at all weeks of CSDS (*p* < 0.05). Water intake was not altered for either group or sex. **Figure 2B** shows weight gain changes in male and female CSDS and controls. In males, there were significant main effects of “Time” (*F*_(3,30)_ = 105.782, *p* < 0.05) and “Stress” (*F*_(1,32)_ = 53.176, *p* < 0.05), and a “Time” × “Stress” interaction (*F*_(3,30)_ = 12.690, *p* < 0.05). In females, there was a main effect of “Time” (*F*_(3,30)_ = 120.861, *p* < 0.05). All pairwise multiple comparisons revealed that CSDS significantly reduced body weight gain in males compared to controls at all weeks of CSDS (*p* < 0.05). **Figure 2C** shows extended non-reproductive days in CSDS females compared to controls (*p* < 0.05). CSDS did not alter CORT levels in either sex (**Figure 2D**).

### Characterization of Behaviors by Intruders during Daily Encounters

**FIGURE 3:**
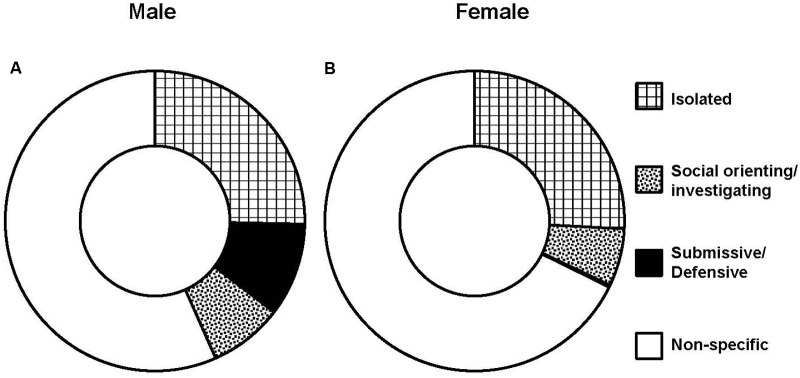
**Behaviors displayed by intruders during daily encounters.** Average of durations by categories in male (*n* = 12) **(A)** and female (*n* = 14) **(B)** intruders.

Male intruders received averages of 9.3 ± 2.13 s of sideways threats and 7.8 ± 1.66 times of attack bites per encounter (5 min maximum) from male “residents” over the course of CSDS. As a result, male intruders spent the longest time on immobile behavior during the encounters, followed by time being sniffed on body and genitalia (**Table 1**). On the other hand, female intruders received 0.1 ± 0.14 s of sideways threats and 0.1 ± 0.1 times of attack bites per 30 min of an encounter by female lactating “residents” over the course of CSDS. As a result, female intruders showed the longest time during encounters on immobile behavior, followed by times on rearing and self-grooming (**Table 1**). **Figure 3** shows pie charts of these behaviors collapsed by categories for both sexes. As expected, both males and females spent the longest time on isolated behaviors. The second longest time spent during the daily encounters was submissive/defensive behaviors for males and social orienting/investigating behaviors for females.

### CSDS Reduces GFAP-IR and GLT-1 Protein Expression

**FIGURE 4:**
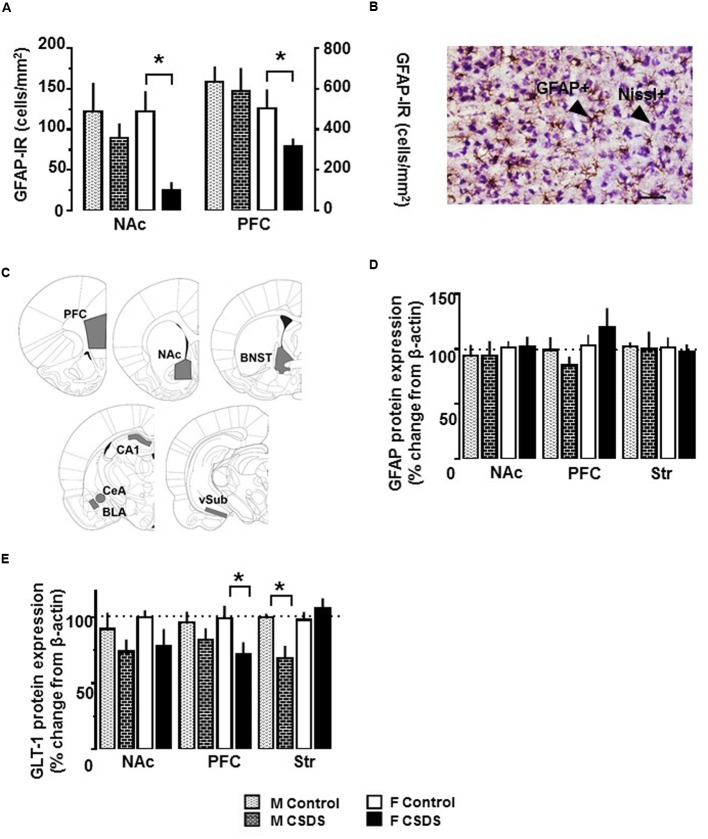
**Immunoreactivity of GFAP-expressing astrocytes, and expression of GFAP and GLT-1 protein. (A)** Density of GFAP-expressing astrocytes (GFAP^+^ cells/mm^2^) in the NAc and PFC in CSDS (male, *n* = 5; female, *n* = 6) and control (male, *n* = 6; female, *n* = 6) groups, as assessed with immunoreactivity to GFAP (GFAP-IR). **(B)** Representative photomicrograph of GFAP-IR and Nissl counterstaining in the PFC in CSDS female. Objective, 10X; scale bar, 50 μm. **(C)** Brain areas used for determining the density of GFAP-expressing astrocytes in CSDS and control groups. PFC, prefrontal cortex (AP +2.7 mm/+2.2 mm); NAc, nucleus accumbens (AP +1.7 mm/+1.2 mm). Protein expression of GFAP **(D)** and GLT-1 **(E)** in the NAc, PFC, and dorsal striatum (Str) in CSDS (male, *n* = 11; female, *n* = 6–8) and control (male, *n* = 7–9; female, *n* = 6–7) groups. Data are expressed as % change from β-actin. Independent *t*-test, ^∗^*p* < 0.05 vs. same sex control. Mean ± SEM.

**Figure 4A** shows density for immunoreactivity to GFAP^+^ cells (GFAP-IR) in the NAc and PFC in both males and females. An independent *t*-test revealed that CSDS reduced the density of GFAP^+^ cells in both structures in females compared to controls (*p* < 0.05). The reduction was not seen in CSDS males. Also, CSDS did not alter GFAP^+^ cells in any other structures tested for either sex (**Table 2**). The entire cell population (Nissl^+^ cell density) was not affected by CSDS for either sex for either region (data not shown). A representative photomicrograph of GFAP^+^ and Nissl^+^ cells and the brain areas selected for the cell counting are shown in **Figures 4B,C**. GFAP protein expressions were not altered in PFC, NAc, and Str for either sex (**Figure 4D**). **Figure 4E** shows GLT-1 protein expression in the NAc, PFC, and the Str. There was a statistical reduction of GLT-1 protein expression in PFC for females [*t*(13) = 2.184, *p* < 0.05] and in the Str for males, compared to their respective controls [*t*(18) = 2.954, *p* < 0.05]. No differences were observed in β-actin protein levels for either group or sex. Representative Western blotting bands for GFAP, GLT-1, and β-actin in the NAc are shown in Supplementary Figures S1A–C.

**Table 2:** Densities of GFAP-expressing (GFAP^+^) cells (GFAP-IR).

	Density of GFAP^+^ cells (cells/mm^2^)			
Brain areas	Male		Female	
	Control	CSDS	Control	CSDS
BNST	670.57 ± 170.23	632.07 ± 176.45	706.22 ± 323.49	383.19 ± 139.26
CeA	162.33 ± 15.68	111.36 ± 47.03	159.42 ± 40.14	107.24 ± 28.72
BLA	319.76 ± 87.75	166.7 ± 67.56	332.44 ± 104.55	166.48 ± 54.39
Dorsal CA1	2904.37 ± 609.19	1842.63 ± 299.74	2120.13 ± 61.45	1752.30 ± 159.03
vSub	315.29 ± 92.98	189.99 ± 46.42	320.96 ± 83.38	168.85 ± 55.4

*BNST, bed nucleus of stria terminalis (AP +0.24 mm/-0.48 mm); CeA, central nucleus of the amygdala; BLA, bed nucleus of the amygdala; CA1, hippocampal CA1 (AP -2.64 mm/-3.24 mm); vSub, ventral Subiculum (AP -4.8 mm/-5.64 mm). CSDS (male, *n* = 5; female, *n* = 6) and control (male, *n* = 6; female, *n* = 6) groups.*

### CSDS Accumulates Glutamate and Reduces Extracellular Glutamine in the NAc

**FIGURE 5:**
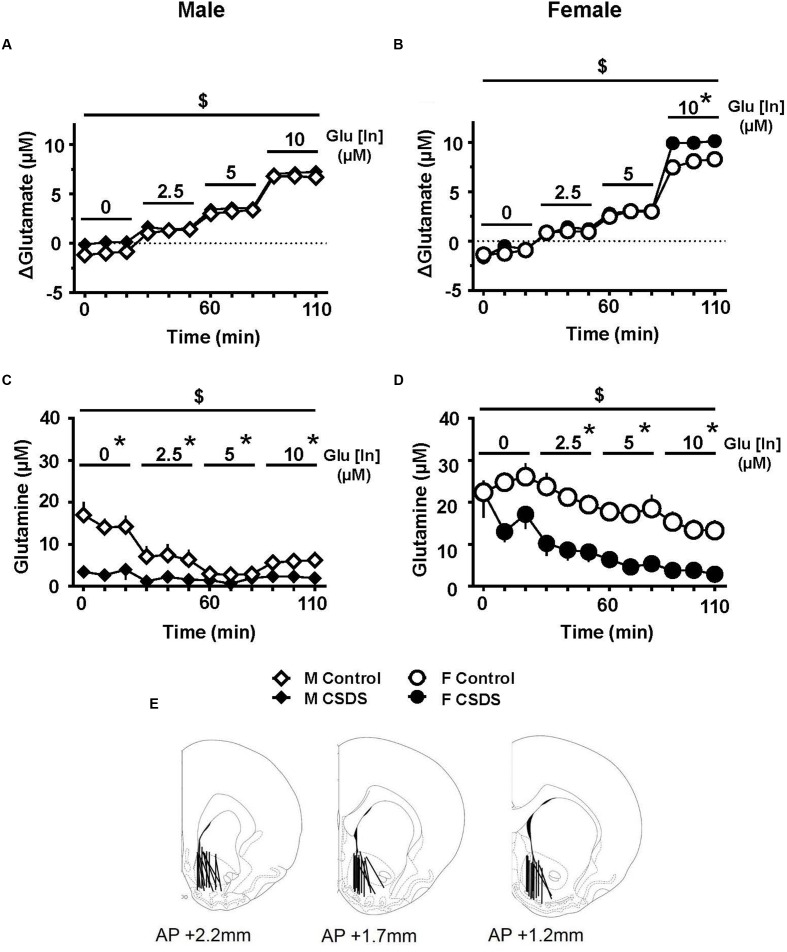
**Glutamate clearance and glutamine release in the NAc. (A,B)** Estimated glutamate accumulation in the NAc (ΔGlutamate, μM) while infusing 0, 2.5, 5.0, and 10.0 μM of glutamate (Glu _[In]_) in males **(A)** (CSDS, *n* = 11; control, *n* = 11) and in females **(B)** (CSDS, *n* = 10; control, *n* = 11). **(C,D)** Extracellular glutamine in the NAc (μM) while infusing 0, 2.5, 5.0, and 10.0 μM of glutamate (Glu _[In]_) in males **(C)** and females **(D)** in CSDS and control groups. Two-way repeated measures of analysis of variance, followed by Holm–Sidak’s *post hoc* test. ^$^Time effect, ^∗^Stress effect. *p* < 0.05. Mean ± SEM. **(E)** Microdialysis probe placements used in the analyses.

**Figures 5A,B** show an estimated accumulation of glutamate (ΔGlutamate) in the NAc while infusing 0, 2.5, 5.0, and 10.0 μM of glutamate (Glu _[In]_) in males and females for CSDS and controls. In females, there was a “Time” effect on ΔGlutamate (*F*_(3,10)_ = 123.308, *p* < 0.05). Pairwise comparisons revealed that CSDS females showed a higher level of ΔGlutamate compared to controls, when 10.0 μM of glutamate was infused_._ In males, there was a significant “Time” effect (*F*_(3,4)_ = 45.688, *p* < 0.05). **Figures 5C,D** show the levels of extracellular glutamine in the NAc while infusing 0, 2.5, 5.0, and 10.0 μM of glutamate (Glu _[In]_) in males and females for both CSDS and controls. There was a significant “Time” effect for both males and females (*F*_(3,8)_ = 17.454, *p* < 0.05 and *F*_(3,7)_ = 16.242, *p* < 0.05, respectively). Pairwise comparisons revealed that both CSDS males and females showed a significant reduction in extracellular glutamine compared to their respective controls, when 2.5, 5.0, and 10 μM of Glu was infused (*p* < 0.05). Moreover, CSDS males had significant lower levels of spontaneous glutamine compared to controls (0 μM of Glu _[In]_) (*p* < 0.05). Estrous phase of all females tested for microdialysis fell into non-reproductive days. The probe placements used in the analysis are depicted in **Figure 5E**.

## Discussion

This study provides evidence that 21 days of territorial or maternal aggression induces depressive-like deficits, and impairs astrocytes in male and female intruders, respectively, as demonstrated by reductions in GFAP-expressing astrocytes and GLT-1 expression in mesocorticolimbic structures and the disruption of the Glu–Gln cycle in the NAc.

Astrocytes in the tripartite synapse are responsible for recycling glutamate. Any insult to astrocytes can disrupt the glutamate homeostasis that leads to glutamate excitotoxicity, which has been demonstrated in many neurodegenerative disorders, and implicated in some psychiatric disturbances in both humans and animal models (Rothstein et al., 1996; Reissner and Kalivas, 2010; Scofield and Kalivas, 2014; Filous and Silver, 2016). Studies in postmortem human depressed subjects and animal models of depression demonstrate reduced immunoreactivity of GFAP (GFAP-IR) and GLT-1 protein expression (Czeh et al., 2006; Banasr and Duman, 2008; Ye et al., 2011; Araya-Callis et al., 2012). In line with others, our female intruders, who were exposed to repeated maternal aggression for 21 days, significantly reduced prefrontal and accumbal GFAP^+^ cells and prefrontal GLT-1. However, our model of depression in males did not show such robust reductions in those structures, particularly for GFAP. One study suggested that chronic stress induces atrophy of astrocytes, as indicated by reduced astrocyte process length, branching, and volume using GFAP as a marker, but did not observe a reduction of the number of astrocytes when cell bodies were counterstained with Nissl (Tynan et al., 2013). In fact, GFAP is a skeleton protein and is not detectably present in cell bodies (Sofroniew and Vinters, 2010; Sofroniew, 2015). Therefore, we only counted GFAP^+^ cells with more than two stem branches extended from a cell body that was counterstained with Nissl. This difference in criteria could be the reason why our depression model in males did not show such robust effects. Also, it should be noted that other markers present in astrocytic cell bodies, such as S-100β or aldehyde dehydrogenase 1L1 (ALDH 1L1), are known to be more specific than GFAP for detecting astrocytes (Cahoy et al., 2008; Hinwood et al., 2013). Therefore, future studies should consider using these markers with respect to astrocytic changes in chronically stressed animals.

In female intruders, while there was a robust reduction of GFAP-IR compared with their respective non-stressed controls, no change was observed in GFAP protein expression as assessed with Western blotting. Although it is counterintuitive, this discrepancy could be due to a methodological difference in sample preparation between immunohistochemistry (IHC) and Western blotting. As described above, we only counted GFAP^+^ cells with more than two stem branches extended from a cell body that was counterstained with Nissl. Hence, it is possible that the fragmented branches of astrocytes were not counted as cells in the IHC assay, but were detected as GFAP protein in Western blotting. In addition, PFA perfusion during the process of brain fixation could have masked epitopes of intracytoplasmic GFAP that prevented antibodies from binding to GFAP (Howat and Wilson, 2014). This interference in binding could significantly affect the detection of GFAP in IHC, as GFAP is only expressed in 15% of a total volume of astrocytes (Bushong et al., 2002).

Glutamate clearance, as determined by the no-net-flux *in vivo* microdialysis, indirectly assesses the functional properties of glutamate transporters through which excess glutamate is eliminated from the ECS (Baker et al., 2002, 2003; Shen et al., 2014). Our results demonstrate for the first time that CSDS accumulates glutamate in the NAc specifically in females when 10 μM of glutamate is infused. The disruption in clearance or kinetics of accumbal glutamate has been associated with a decrease in astrocytic GLT-1 (Knackstedt et al., 2010; Shen et al., 2014). In the NAc, while the density of GFAP-IR was significantly reduced, the protein expression of GLT-1 was not altered in the CSDS groups in our study. Because GLT-1 is primarily expressed in astrocytes (Anderson and Swanson, 2000), it is counterintuitive that the decrease of GFAP-IR was not reflected in the expression of GLT-1, particularly for females. One classical explanation would be estrogens. Estrogens are known to facilitate GLT-1 promotor activity to restore GLT-1 function (Pawlak et al., 2005a; Lee et al., 2012, 2013). In our study, however, CSDS extended the non-reproductive days, indicating low circulating estrogens. The estrous phase of the day of sample collection for all females was in non-reproductive phases. In addition, we see a clear stress-induced decrease of GLT-1 in the PFC, and it is unlikely that the estrogens had a local effect on preventing the GLT-1 impairment only in the NAc. Rather, it is possible that the sample size for the NAc of each rat was insufficient to detect the stress effect. As demonstrated by Rothstein et al. (1994), NAc expresses relatively low GLT-1 compared to other brain structures, such as the cortex. Also, one study demonstrated that internalization of GLT-1 from the plasma membrane can impair glutamate uptake without altering the total protein levels of GLT-1 (Nakagawa et al., 2008). Therefore, it is not surprising that the effect of CSDS was not apparent on the changes of GLT-1 expression in the NAc.

To our knowledge, this is the first study to highlight the effect of CSDS on extracellular glutamine in the NAc. Glutamine is primarily synthesized from glutamate transferred from ECS to astrocytes via GLT-1. In both males and females, CSDS significantly reduced the extracellular glutamine when glutamate was infused in a range of 2.5–10 μM. Extracellular glutamine can be referred to as a gliotransmitter (Halassa et al., 2007); therefore, the reduction of glutamine seen in our study is possibly due to reduced astrocytes in the NAc. However, given the fact that we did not see a clear reduction of GLT-1 in the NAc for either CSDS males or females, there is also a possibility that the reduction of extracellular glutamine is not dependent on astrocytes or GLT-1. Future assessments should focus on the role of glutamine on CSDS and associated depressive-like deficits involving other cell types or transporters.

Chronic social defeat stress has construct, predictive, and face validity as a depression model, and is widely used in studying the neurobiology of depression (Miczek et al., 2008; Rygula et al., 2008). However, using this model for sex comparison studies of depression has been a challenge, since the ethological procedure by which social defeat is arranged is distinctive between males and females. Generally, in laboratory rodents, male social defeat refers to a submission of territorial aggression composed of highly organized and offensive behaviors displayed by the “residents” (Crawley and Contrera, 1976; Murphy, 1976; Miczek, 1978). In contrast to males, female “residents” typically do not display territorial aggression toward intruders, except for specific species (Trainor et al., 2011; Laredo et al., 2015). Hence, female social defeat usually refers to maternal aggression by conspecific lactating dams showing both offensive and defensive aggression (Erskine et al., 1978; Lucion and De Almeida, 1996; Shimamoto et al., 2011, 2014; Holly et al., 2012). Consistent with others, we show that males receiving chronic territorial aggression spend a significant time on submissive/defensive behaviors, as a result of receiving intense offensive attacks as indicated by sideways threats and attack bites. We show for the first time that females spend a significant time on isolated behaviors, and a very short time on submissive/defensive behaviors while receiving daily maternal aggression. This modest expression of submissive/defensive behaviors in female intruders contrasts with our previous observations (Shimamoto et al., 2011). The reason for this difference in the expression of submissive/defensive behaviors as a result of maternal aggression is not clear, but could be the result of differences in the protocol environment. Moreover, in the present study, we show that repeated maternal aggression may be inducing some levels of anxiety-like behaviors in female intruders, as indicated by the ample times spent on rearing and self-grooming. This is in line with other studies on females repeatedly receiving other types of stressors (Bourke and Neigh, 2012; Bowman and Kelly, 2012; Ver Hoeve et al., 2013). Given that both males and females showed similar reductions in saccharin intake, it is unlikely that the differential sex impairments in GFAP, GLT-1, and glutamate accumulation or glutamine release are simply due to the differential aggression used to produce the depression models. However, when using the CSDS protocols, full disclosure of precise assessments related to defeated behaviors for both males and females is still warranted.

A dysregulated hypothalamic–pituitary–adrenal (HPA) axis has been well-documented in some stress-related disorders and can be a biological marker for assessing a depressive-like phenotype in animals (Checkley, 1996; Heim et al., 2000). While our study showed a robust anhedonia-like responses in both male and female intruders, as indicated by prolonged suppression in saccharin intake compared with their respective controls, these intruders did not show altered CORT levels. Studies across the literature point out that circulating CORT levels, as well as CORT responses to acute stressors, can vary for chronically stressed animals in both sexes (Haller et al., 1998; Papaioannou et al., 2002; Dalla et al., 2005; Bowman et al., 2006; Krishnan et al., 2007; Mathews et al., 2008; Reich et al., 2009; Bourke and Neigh, 2011; Hodes et al., 2015). Thus, it is not surprising that we did not see a robust response on the HPA axis. Rather, we and others have shown that the depression model disrupts the estrous cycle in females (Dalla et al., 2005; Baker and Bielajew, 2007; Shimamoto et al., 2011, 2014). Therefore, when modeling human depression, tracking the estrous cycle can be an appropriate tool to enhance the validity of modeling depression in females, in addition to anhedonia-like responses.

## Conclusion

Twenty one days of territorial or maternal aggression induced depressive-like deficits and impaired astrocytes in both male and female intruders. Female intruders had reduced prefrontal and accumbal GFAP-IR and prefrontal GLT-1. These females showed disrupted glutamate clearance when a high concentration of glutamate was infused into the NAc. Male intruders had reduced striatal GLT-1 and extracellular glutamine in the NAc. The disruption of the glutamate–glutamine cycle in the PFC-striatal network, including the NAc, may be linked to depression-like symptoms more so in females than in males.

## Author Contributions

VR executed experiments on CSDS, *in vivo* microdialysis, GFAP-IR, and measured behaviors. AB performed experiments on Western blotting for GFAP and GLT-1. JP conducted experiments on HPLC and CORT assay, and assisted VR on CSDS, GFAP-IR, and behavioral analyses. BY provided financial support on Western blotting for GFAP and GLT-1. AS provided the experimental design, and conducted experiments on *in vivo* microdialysis and HPLC, and trained VR and JP for CSDS, *in vivo* microdialysis, and HPLC. VR and AS performed all the statistical analyses and wrote the manuscript.

## Conflict of Interest Statement

The authors declare that the research was conducted in the absence of any commercial or financial relationships that could be construed as a potential conflict of interest.

## Footnotes

1 https://www.eicomusa.com/hplc-ecd/applications/glutamate-gaba-12-minute-analysis/
