# Supplementary figures and images for: Disruption of the Glutamate–Glutamine Cycle Involving Astrocytes in an Animal Model of Depression for Males and Females
Source: Front Behav Neurosci. 2016 Dec 6;10:231. doi: 10.3389/fnbeh.2016.00231 (PMC5147055; doi:10.3389/fnbeh.2016.00231)

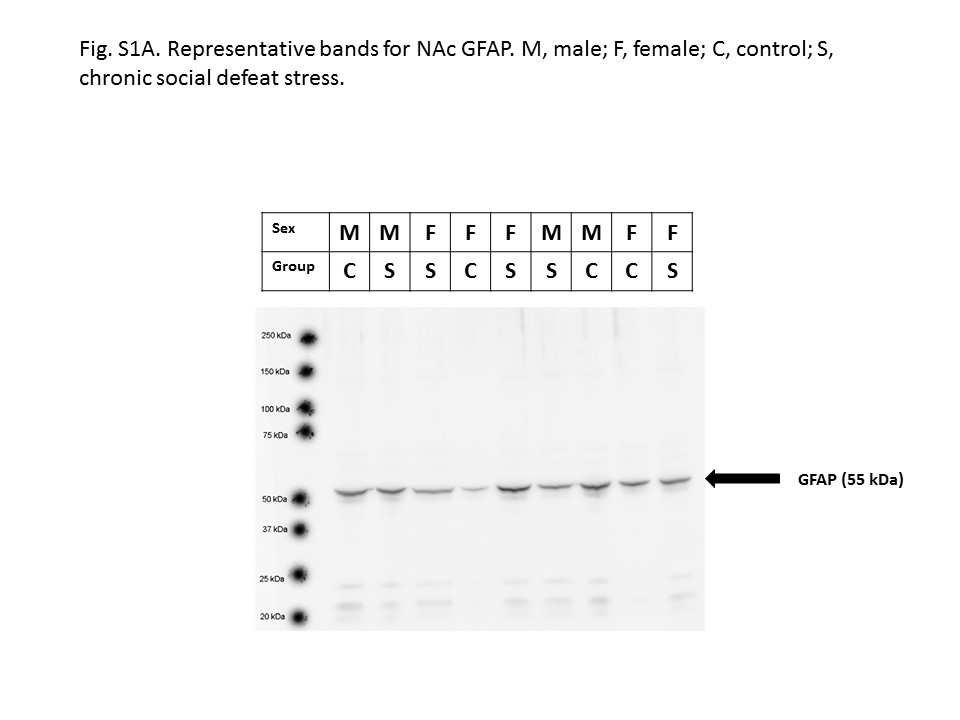

Supplement: Supplementary file 1 [file Image_1.JPEG]

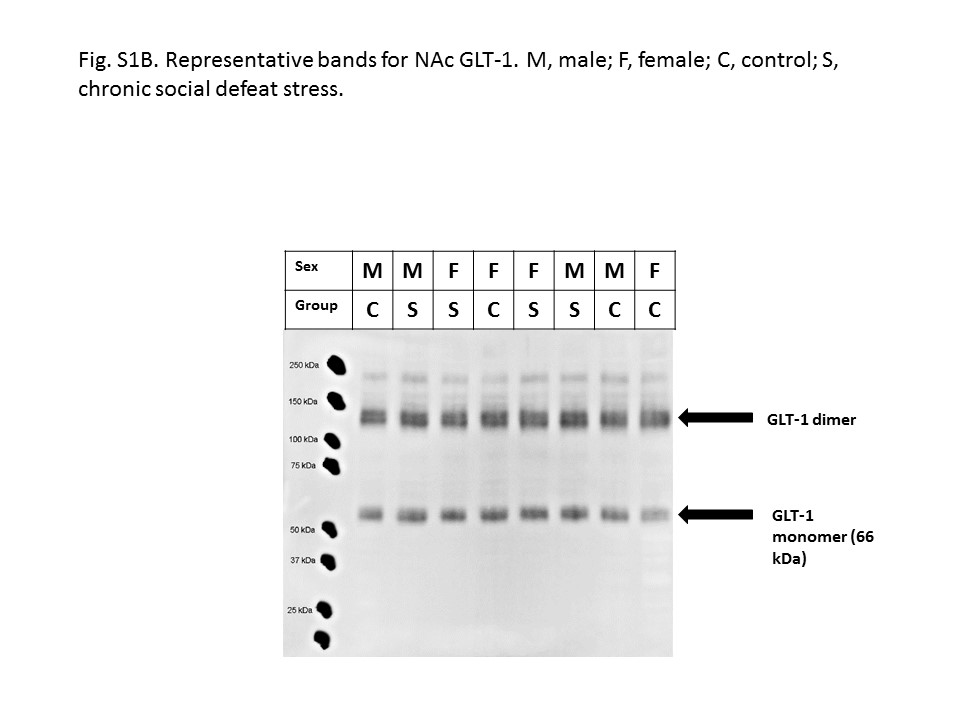

Supplement: Supplementary file 2 [file Image_2.JPEG]

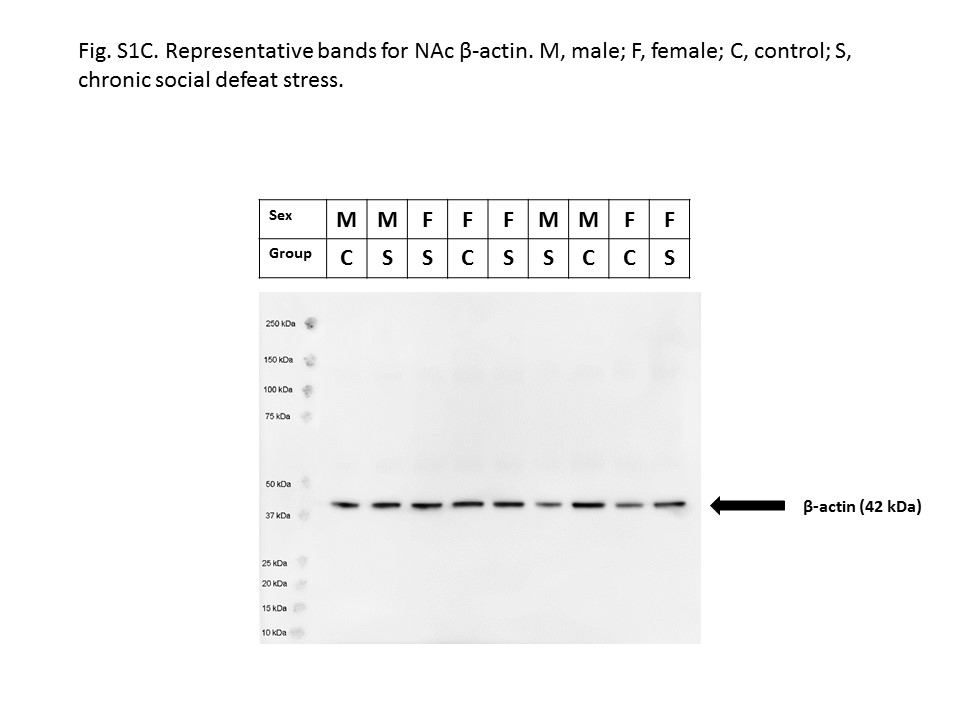

Supplement: Supplementary file 3 [file Image_3.JPEG]
