# Supplementary material for: Disruption of the Glutamate–Glutamine Cycle Involving Astrocytes in an Animal Model of Depression for Males and Females
Source: Front Behav Neurosci. 2016 Dec 6;10:231. doi: 10.3389/fnbeh.2016.00231 (PMC5147055; doi:10.3389/fnbeh.2016.00231)
Supplement: Supplementary file 4 [file Table_1.DOCX]

| **Experiments** | **Sex** | **Group** | **N** | **Analysis** | **Corresponding Fig.** |
| --- | --- | --- | --- | --- | --- |
| Saccharin test | M | CTRL | 15 | Two-Way Repeated Measure ANOVA | Fig. 2A |
|  | M | CSDS | 19 |  | Fig. 2A |
|  | F | CTRL | 18 | Two-Way Repeated Measure ANOVA | Fig. 2A |
|  | F | CSDS | 16 |  | Fig. 2A |
| Weight | M | CTRL | 15 | Two-Way Repeated Measure ANOVA | Fig. 2B |
|  | M | CSDS | 19 |  | Fig. 2B |
|  | F | CTRL | 18 | Two-Way Repeated Measure ANOVA | Fig. 2B |
|  | F | CSDS | 16 |  | Fig. 2B |
| Estrous cycle | F | CTRL | 18 | Independent t-test | Fig. 2C |
|  | F | CSDS | 16 |  | Fig. 2C |
| CORT | M | CTRL | 4 | Independent t-test | Fig. 2D |
|  | M | CSDS | 4 |  | Fig. 2D |
|  | F | CTRL | 4 | Independent t-test | Fig. 2D |
|  | F | CSDS | 7 |  | Fig. 2D |

Supplementary Table S1. Statistical analyses and numbers used in the study (continued to next page).

Table S1. Statistical analyses and numbers used in the study (continued from previous page).

| **Experiments** | **Sex** | **Group** | **Numbers** | **Analysis** | **Corresponding Fig.** |
| --- | --- | --- | --- | --- | --- |
| Immunohistochemistry | M | CTRL | 6 | Independent t-test | Fig. 4A |
|  | M | CSDS | 5 |  | Fig. 4A |
|  | F | CTRL | 6 | Independent t-test | Fig. 4A |
|  | F | CSDS | 6 |  | Fig. 4A |
| Western blot | M | CTRL | 7-9 | Independent t-test | Fig. 4D and E |
|  | M | CSDS | 11 |  | Fig. 4D and E |
|  | F | CTRL | 6-7 | Independent t-test | Fig. 4D and E |
|  | F | CSDS | 6-8 |  | Fig. 4D and E |
| No-net-flux *in vivo* microdialysis | M | CTRL | 11 | Two-Way Repeated Measure ANOVA | Fig. 5A and C |
|  | M | CSDS | 11 |  | Fig. 5A and C |
|  | F | CTRL | 11 | Two-Way Repeated Measure ANOVA | Fig. 5B and D |
|  | F | CSDS | 10 |  | Fig. 5B and D |
